# Degeneracy and Complexity in Neuro-Behavioral Correlates of Team Coordination

**DOI:** 10.3389/fnhum.2020.00328

**Published:** 2020-09-10

**Authors:** Silke Dodel, Emmanuelle Tognoli, J. A. Scott Kelso

**Affiliations:** ^1^Center for Complex Systems and Brain Sciences, Florida Atlantic University, Boca Raton, FL, United States; ^2^Intelligent Systems Research Centre, University of Ulster, Derry∼Londonderry, United Kingdom

**Keywords:** social coordination, hyperscanning, degeneracy, manifold, coherence, symmetry breaking, team work, wavelets

## Abstract

Team coordination—members of a group acting together rather than performing specific actions individually—is essential for success in many real-world tasks such as military missions, sports, workplace, or school interactions. However, team coordination is highly variable, which is one reason why its underlying neural processes are largely unknown. Here we used dual electroencephalography (EEG) in dyads to study the neurobehavioral dynamics of team coordination in an ecologically valid task that places intensive demands on joint performance. We present a novel conceptual framework to interpret neurobehavioral variability in terms of *degeneracy*, a fundamental property of complex biological systems said to enhance flexibility and robustness. We characterize degeneracy conceptually in terms of a manifold representing the geometric locus of the dynamics in the high dimensional state-space of neurobehavioral signals. The geometry and dimensionality of the manifold are determined by task constraints and team coordination requirements which restrict the manifold to trajectories that are conducive to successful task performance. Our results indicate that team coordination is associated with dimensionality reduction of the manifold as evident in increased inter-brain phase coherence of beta and gamma rhythms during critical phases of task performance where subjects exchange information. Team coordination was also found to affect the shape of the manifold manifested as a symmetry breaking of centro-parietal wavelet power patterns across subjects in trials with high team coordination. These results open a conceptual and empirical path to identifying the mechanisms underlying team performance in complex tasks.

## 1. Introduction

Success in real-world tasks is crucially dependent on the quality of team efforts. Performing a task as a team requires that team members mutually coordinate their actions. Irrespective of the skills that team members have for performing certain actions, it is their coordination that distinguishes high team performance from the less desirable outcome achieved when the very same actions are performed incoherently by its members. Such is the notorious problem of people-matching that pervades many human activities. Although multiple behavioral studies of team coordination have been conducted (Fiore et al., 2003; Cooke et al., 2007; Woolley et al., 2007; Salas et al., 2008; Astolfi et al., 2010; Bourbousson et al., 2010; DeChurch and Mesmer-Magnus, 2010; Dodel et al., 2010; Gorman et al., 2010; Gorman and Cooke, 2011; Stevens et al., 2016; Nordham et al., 2018), the underlying neural processes are currently still largely unknown (Astolfi et al., 2010; Dodel et al., 2011; Stevens et al., 2011; Tognoli et al., 2011b; Cooke et al., 2012; Likens et al., 2014). Team coordination implies that team members mutually adapt their behavior in anticipation of, and in response to, the behavior of their teammates. Variability is thus both a source and a consequence of effective team coordination. Typically, team performance is contingent on the ability of the team members to compensate and even exploit behavioral variability of themselves and the other(s). Variability of behavioral and brain dynamics is an integral aspect of real world and ecologically valid laboratory tasks, since teammates have to adapt to a changing context, sometimes in a very subtle way. Here we argue that such ubiquitous variability is a reflection of degeneracy, a fundamental principle of nature, which endows biological systems with flexibility and robustness (Edelman and Gally, 2001). On an abstract level degeneracy signifies the ability of elements that are structurally different to perform the same function (Tononi et al., 1999; Greenspan, 2012; Kelso, 2012) (see Tieri et al., 2010 for a history of the term degeneracy and Kelso et al., 1980a for a specific example in the field of motor control). Hence to study team coordination, it is imperative to take degeneracy into account. In the following we present a conceptual framework for the analysis of jointly performed tasks that models degeneracy and interprets it as an integral part of task performance rather than as a hindrance as in methodologies that focus on a single path to task performance. We deploy this framework in conjunction with dual electroencephalography (EEG) to identify neural signatures of team coordination in human dyads. The paper is organized as follows: We first describe the EEG experiment (section 2.1) and the behavioral segmentation of the task (section 2.2). Then we present the conceptual framework for behavioral dynamics (section 2.3) and brain dynamics (section 2.4) and how they are related. In section 2.5, we discuss team coordination as a measure of team performance and we explain how to use our conceptual framework in this context. In sections 2.7 and 2.8, we introduce two ways of applying the conceptual framework to the EEG data from our experiment, the results of which are presented in sections 3.1 and 3.2. Finally in section 4, we discuss our results and the overall framework in a broader context.

## 2. Materials and Methods

### 2.1. Dual EEG Experiment

**Figure 1:**
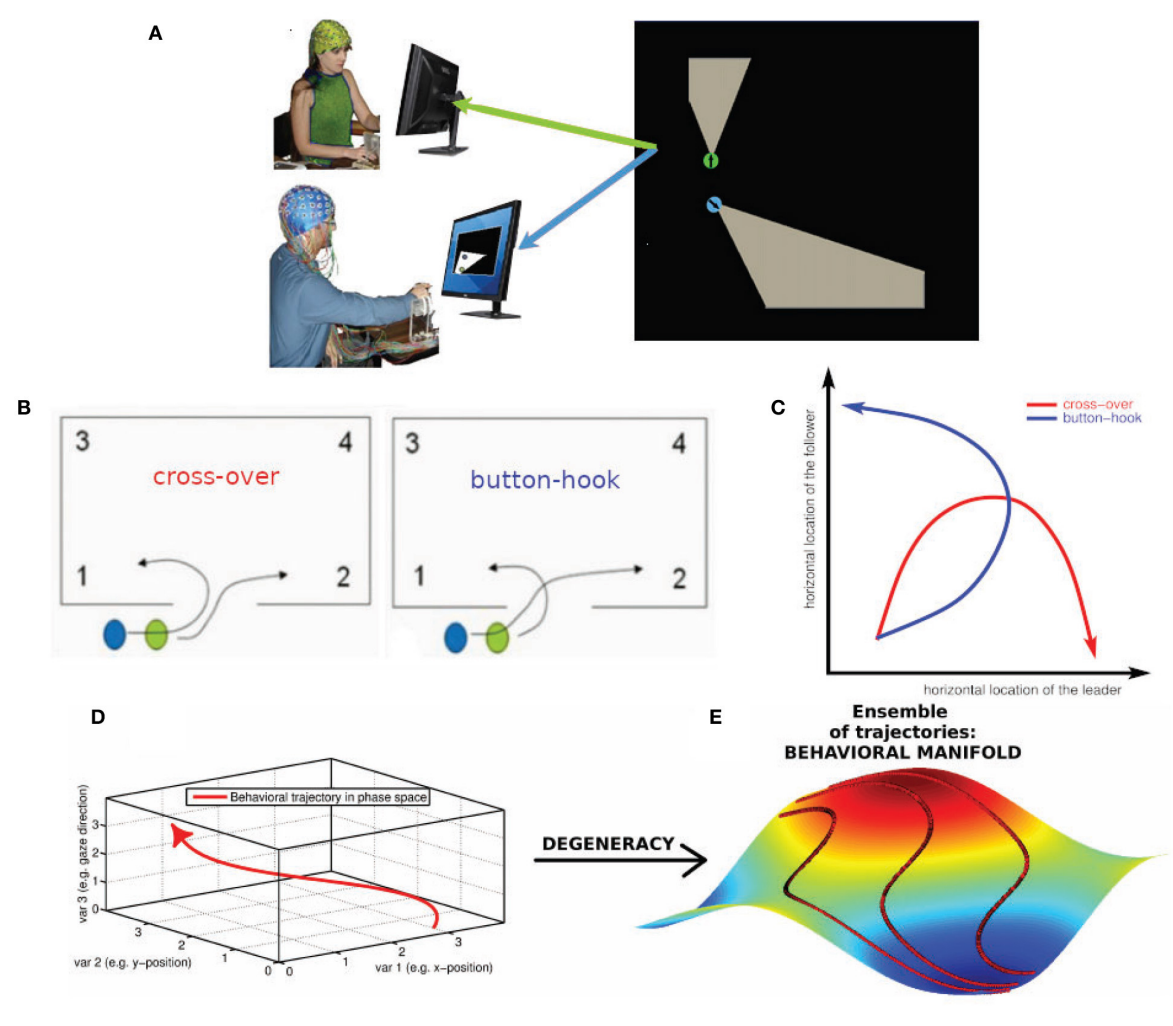
Degeneracy and the geometrical description of behavior. **(A)** Human dyad engaged in the virtual room clearing task **(B)** Behavioral degeneracy: Two qualitatively different entry patterns: Leader cross-over (left) and leader button-hook (right). Leader (green) and follower (blue). Both entry patterns are valid forms of task execution. **(C)** Illustration of the degeneracy of the entry pattern in a 2-D state space comprised of the horizontal locations of the leader and follower, respectively. In the cross-over entry the leader's horizontal location (x-axis) increases while the follower's horizontal location (y-axis) first increases and then decreases (red curve). The converse is the case for the button-hook entry (blue curve) **(D)** Geometrical description of a behavior as a trajectory in a high-dimensional state space visualized as 3-dimensional space. **(E)** Degeneracy leads to an ensemble of potential behavioral trajectories which together form a manifold. The 3D manifold (colored surface) and three sample trajectories (red tracks) illustrate the concept. Note that in most cases the behavioral state space has a dimension greater than three and therefore the behavioral manifold cannot be easily visualized. In our case the behavioral state-space of the avatars is 4-dimensional (2-D coordinates of the two avatars) or 6-dimensional, if the respective pieing angles of the two avatars are taken into account as well.

We measured dual EEG in dyadic teams who performed in a computer simulation of a room clearing task (Tognoli et al., 2011a). Room clearing is an operation in which teams move through a building room-by-room in a highly codified manner in order to neutralize possible threats encountered from hostile others at the same time as protecting each other. Room clearing requires one of the most extreme forms of team coordination in that survival and safety of the team members depend on efficient team interactions and communication. The computerized task was designed to retain key psychological processes that occur in successful team work. Behavioral sequences followed the main lines of an instructional video of room clearing developed under the US Office of Naval Research's VIRTE program at Clemson University (details in Tognoli et al., 2011a). During the task, each subject sat in front of a computer screen and had a top down perspective of the virtual environment (Figure 1A), including their and their partner's avatar, outlines of walls and doors, and enemies, which were present with a probability of 0.5. Avatar position and direction of gaze were operated with a gaming (Xbox) controller, the use of which was familiar to all subjects. Subjects navigated their avatars through a series of 32 buildings (five successive rooms each). Their shared virtual environment became visible upon the avatars' spatial exploration (cone of vision of 45 degrees for each). We measured dual EEG from 16 dyads (32 subjects). Two 60-channel arrays (1 per subject, impedances below 10 KΩ) were simultaneously fed to a single EEG amplifier for time accuracy, and the signals (references to linked mastoids, ground at FPz) were differentially amplified, filtered (0.1–200 Hz) and digitized (1,000 Hz) at 24 bit resolution. Electro-ocular activities were also collected to identify blinks and saccades, and shielding and guarding ensured minimal contamination from noise-emitting equipment. For additional information, please refer to Tognoli et al. (2011b). The analyses described below were done on artifact corrected EEG. For artifact corrections specific to this work, we identified epochs contaminated with ocular blinks using a windowed linear approximation over EOG channels to identify peaks; rejected muscular artifacts by thresholding wavelet power in the band (40–60 Hz); and removed linear trends.

### 2.2. Behavioral Segmentation of Virtual Room Clearing Task

The room clearing task has a specific temporal structure that requires members to alternate between collective and independent behavior as a premise for performance. Each room clearing trial involves three stages: the building up, sustaining, and breaking up of interpersonal coordination (Tognoli et al., 2011a) (see also Oullier et al., 2008 for a related construct). The building up of coordination occurs both before the entry as well as at the end of the room clearing task. Table 1 gives a behavioral segmentation of the stages of the virtual room clearing task and the associated information flow between the subjects. The stages of coordination are as follows: (1) coordination build-up while stacking in front of the room (segments s0 and s1), (2) coordinated behavior when moving to the door and entering the room (segments s2–s4), (3) breaking of coordinated behavior (segments s5–s7a), and (4) rebuilding of coordination (s7b–s9). Each room clearing trial proceeds as follows: Before each room entry, team members spontaneously organize their respective roles of leader and follower (segment s9). The leader is the one whose avatar is closest to the door and enters the room first. The follower initiates the maneuver by signaling to the leader to start moving (segment s0), in real life with a tap on the shoulder and in the computerized task through a button press on his or her Xbox controller that results in a short vibration of the leader's Xbox controller (segment s1). In order to minimize EEG artifacts, verbal and gestural communication were not allowed. Upon the follower's signal the leader initiates motion of his avatar (segment s2) and is followed by the avatar of the follower. Then both subjects move their avatars to the door as close as possible and the leader presses a button to open the door (segment s3). The leader enters the room, followed as closely as possible by the follower (segment s4). Then both leader and follower independently move to their respective “corners of dominance,” strategic positions which are the two room corners adjacent to the door (segment 5). Both corners of dominance must be covered, hence the follower needs to direct his/her avatar to the corner opposite from the leader's. Immediately after entering and while moving toward the corners of dominance, the subjects start “pieing” (rotating their gaze at an increasing angle with respect to their avatar's body motion direction, to scan the room and detect/eliminate threat). To keep subjects alert, there occasionally were enemies or friendly avatars in the room, upon detection of which the subjects had to make a decision to shoot or not to shoot. After arrival at their respective corners of dominance, the subjects continued to pie (segment s6) and had instruction to engage in gaze interlock, i.e., the overlap of their view cones (segment s7a). The gaze interlock signaled complete coverage of the visual scanning by the dyad and ended the trial, with subjects moving to the next stacking point (segment s8).

**Table 1:** Stages of coordination: Task segmentation and associated information flow between the members of the dyad.

		**Task segments**	**Information flow**	
			**Leader**	**Follower**
**Stages of coordination**	Building-up	**Segment s0:**		
		Preparation to tap	↔	
		**Segment s1:**		
		Tap onset follower to movement onset leader	←	
		**Segment s2:**		
		Movement onset leader to movement onset follower	→	
	Maintaining	**Segment s3:**		
		Movement of both subjects to door as close as possible	↔	
		**Segment s4:**		
		Door opening and begin entry pattern by leader, entry of follower	→	
	Breaking-up	**Segment s5:**		
		Movement of both subjects to the corners of dominance.	↮	
		**Segment s6:**		
		Start pieing	↮	
		**Segment s7:**	↮	
		(a) Reach the corner of dominance		
	Building-up	(b) Gaze interlock	↔	
		**Segment s8:**		
		Movement to next stacking point (right or left from next door).	↔	
		**Segment s9:**		
		Restacking (follower's avatar aligns behind leader's avatar).	→	

### 2.3. The Geometry of Behavior

The complex behaviors manifested in this task are embedded in a dataset that already manifests enormous complexity and degeneracy, even as it only stores a fraction of real life behavior. This is readily observed from the fact that no two avatar trajectories by multiple dyads traversing the same room have much overlap but for the strategic points built into the task (near the doors and at the corners of domination). In the following we describe the conceptual framework that we developed to interpret behavioral and brain dynamics in the presence of degeneracy. We first focus on the behavioral level, then on the neural level and then show how the two are linked conceptually. Our aim is to show how degeneracy of brain dynamics can be harnessed to reveal neural correlates of inter-personal coordination.

Performing a task implies that the subjects restrict their behavioral dynamics to meet the requirements of successful task execution. However, the restrictions imposed by the task are in most, if not all cases, insufficient to single out a uniquely optimal behavioral trajectory (Liu et al., 2019). As a concrete instance, in the room clearing task there are two qualitatively different entry patterns called cross-over and button-hook (cf. Figure 1B, Tognoli et al., 2011a) which both represent a valid form of task execution. Even within a given entry pattern, behavioral dynamics may vary to some extent, such as in location, velocity or gaze direction—all without detrimental effect on task performance. This “allowed” variability is an example of behavioral degeneracy, i.e., the ability of different behaviors to execute the same task. Behavioral degeneracy contributes in an essential manner to team coordination, for instance by enabling the follower to select his or her behavior in response to the behavior of the leader in order to correctly execute the task. From the perspective of Coordination Dynamics (e.g. Fuchs and Kelso, 2018; Kelso, 1995, 2009/2013), behavior can be described as occurring in a high-dimensional state space in which each dimension corresponds to a degree of freedom or, more often, a variable of interest that implicitly combines some degrees of freedom from a lower level of description.

Location variables, for instance, are an obvious choice for spanning a behavioral state space. Figure 1C illustrates the degeneracy of the two allowed entry patterns, cross-over and button-hook in a 2-D phase space comprised of the horizontal locations of the leader and follower, respectively. Taking both location coordinates for the two avatars into account, the state space becomes 4-dimensional, adding the direction of gaze increases the dimensionality of the state space to 6-D (Dodel et al., 2013), etc. We note that the choice of variables is determined by the available measurements and the research question of interest. Distinct choices of variables span different state spaces that provide various windows into the problem under consideration. Under a state space representation, a task can be interpreted as an (implicit) set of conditions on the degrees of freedom of the task executing system (here the human dyad) restricting the behavioral dynamics to a subset of configurations (see also Saltzman and Kelso, 1987). The behavioral dynamics in each trial is then represented by a trajectory through the state space. The trajectories do not fill the n-dimensional state space. They represent information about external, internal and mutual constraints in the dyad's coordination dynamics, therefore limiting the relative values that each variable can adopt in the context of each other. External constraints include the avoidance of physical objects and other task requirements. Internal constraints may stem from limits of processing capacity of the subjects, e.g., attentional limitations during the entry phase may impede a subject from simultaneously engaging in “pieing” (scanning the environment with one's gaze in a rotational pattern). Mutual constraints result from the requirement of coordinated behavior between the members of the dyad. The ensemble of all potential state space trajectories forms a flow that occurs on a manifold (Pillai, 2008; Pillai and Jirsa, 2017; Tsuda, 2018) which is the geometric locus of the entire behavioral dynamics in state space that is associated with a given task. The extent of the manifold is related to the variability of the dynamics and the geometry of the manifold reflects the above-mentioned constraints under which the dynamics take place. In short, the manifold is a manifestation of behavioral degeneracy (Scholz and Schöner, 1999). Degeneracy reflects the capacity of systems with a high number of degrees of freedom to perform the same task equally well by using a variety of behaviors. Degeneracy is a concept that relates the dynamics of a system with the concept of a task. The task has the effect of restricting the “admissible” dynamics to only part of the state space. In our case the “admissible” dynamics can be represented by inducing one or more coupling, inducing couplings between the degrees of freedom (the variables of the state space). If the constraints imposed by the task are less than the degrees of freedom there are in general infinitely many trajectories that are compatible with the task. The closer the number of constraints to the number of degrees of freedom of the system, the more restricted the admissible task dynamics (e.g., in synchronized swimming or ballet) (Fuchs and Kelso, 2018). In movement science, degeneracy is conceptualized as motor equivalence (Kelso and Tuller, 1984). It emphasizes that successful task execution can be achieved by the system dynamically forming synergies (groups of degrees of freedom which exhibit coordinated behavior). One reason for this phenomenon is that the degrees of freedom by far outnumber the constraints that are imposed by the task and hence many degrees of freedom remain unconstrained. We posit that degeneracy is generic and occurs in most if not all systems that possess a high number of degrees of freedom. The conceptual and the geometrical view of degeneracy we adopt here are related as follows:

Conceptual view:

variability + constraints + dynamics → degeneracy

Geometrical view:

manifold in state space + geometry + trajectories → flow on manifolds

Figures 1B–E, show the conceptual path from behavior to behavioral manifold. The coupling of degrees of freedom induced by task constraints (external and internal) leads to the manifold being lower dimensional than the state space. In a team task, manifold geometry is also influenced by team performance and inter-subject coordination which are instances of mutual task constraints. These factors affect the shape and further restrict the dimensionality of the manifold. The state space of the behavioral dynamics of multiple subjects is the Cartesian product (i.e., the combination) of the state spaces of the individual behavioral dynamics. However, while a manifold representing independent individual behavior of the team members is simply the Cartesian product of the manifolds of individual behavior, this is not the case for team behavior. In the case of team behavior, the coupling between individual behavioral dynamics induced by inter-subject coordination leads to an inseparability of the manifold representing the behavioral dynamics of the team. As an illustration take two people that walk along a narrow hallway (a one-dimensional structure). Their individual state spaces are line segments while their team state space is a square. Uncoordinated behavior would lead to team trajectories filling the square (assuming one person can overtake the other) while coordinated behavior such as one person closely following the other would narrow down the behavioral manifold of the team to a narrow strip along the diagonal of the square. In summary, the behavioral manifold in the state space of the dyad inherits its geometry from coordinative coupling of degrees of freedom, mediated by task constraints, team performance, and intra- as well as inter-subject coordination.

### 2.4. The Geometry of Brain Dynamics

**Figure 2:**
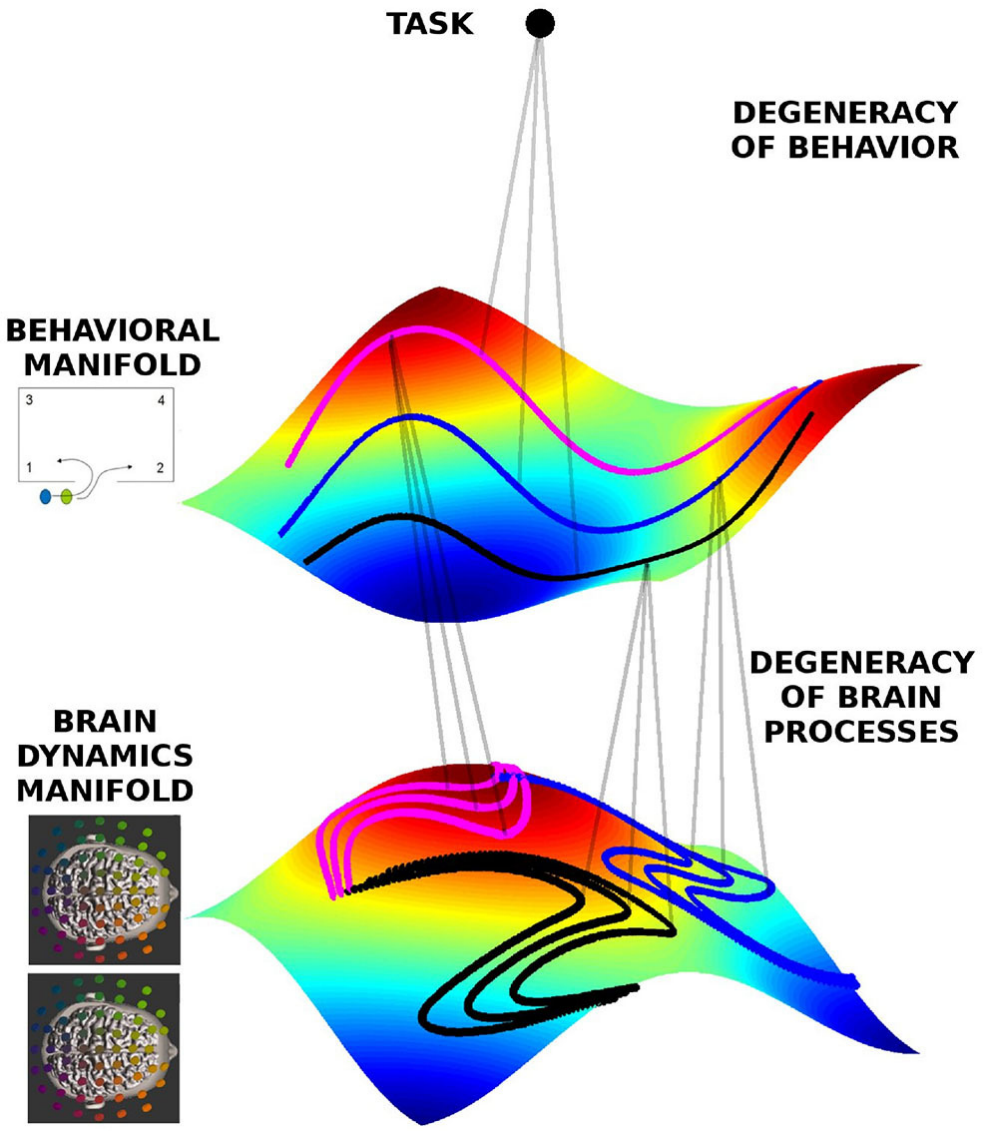
Geometrical framework to formalize degeneracy in behavioral and brain dynamics. The manifolds are represented by the colored surfaces in a 3D state-space (reduced for visualization). All trajectories live on those manifolds, and three exemplars are indicated by the doted lines colored pink, black, and blue, each corresponding to different behavioral dynamics, respectively, but at the same level of team performance, hence lying on the same behavioral manifold. The same behavior can be represented by different neural dynamics, hence on the brain dynamics manifold there are different instances of neural dynamics for each behavioral trajectory.

As exemplified above, behavioral degeneracy refers to a situation where the same task can be accomplished by distinct behaviors. Degeneracy exists also at the neural level. Here degeneracy refers to the fact that the same brain function can be supported by different neural networks (Noppeney et al., 2006; Kelso, 2012; Marrelec et al., 2016), hence the same behavior may be supported by distinct brain processes. This well-known property remains to be integrated in current studies of brain function. Degeneracy is a necessary property of neural processes for several reasons. For one, without degeneracy the brain would not be able to function, because neural plasticity and neuronal death constantly change the network constituting the brain (Tononi et al., 1999; Edelman and Gally, 2001; Bressler and Tognoli, 2006; Edelman, 1978; Kelso and Tuller, 1984). The brain hence needs to remain functional despite its continuously changing material substrate. Furthermore, the brain is an embodied entity living in an ever-changing environment. Hence the context in which neural processes occur is never identical, not even in laboratory conditions (Gallego et al., 2017; Bressler and Kelso, 2001). Moreover, because every time a task is executed, the environmental and neural context is different, the subject must be able to initiate the appropriate behavior and brain activity from different starting points. To account for degeneracy at the neural level we define a brain dynamics state space in analogy to the behavioral state space discussed above. The state space of brain dynamics is spanned by neural variables of interest, such as the EEG power or coherence in various frequency bands. Each brain process of the dyad is represented by a trajectory in the state space of brain dynamics. Figure 2 shows the conceptual relationship between behavioral and brain dynamic manifolds. Inheriting behavioral degeneracy, the ensemble of trajectories in brain dynamics' state space lies on a manifold. However, brain dynamics have an additional source of degeneracy due to the fact that different network connectivities and dynamics are able to support the same brain function. Hence each trajectory in behavioral state space is associated with a part of the brain dynamics manifold comprising all potential trajectories in brain state space that support this behavior. Therefore, in our framework each task is associated with both a behavioral and a brain dynamics manifold which are mutually related (Schöner and Kelso, 1988; Kelso et al., 1998; Fuchs et al., 2000; Pillai and Jirsa, 2017).

### 2.5. Measuring Team Coordination

In real-world applications it is often important to distinguish high from low team performance. This can be done in our framework by comparing the properties of manifolds corresponding to different levels of team performance (Dodel et al., 2010, 2011). Team performance can be integrated into the conceptual picture as an additional axis to the state space along which the manifolds vary. Here we were particularly interested in team coordination as a measure of team performance. The length of the time interval between the follower signaling the leader and the leader initiating his or her avatar's movement is inversely related to leader readiness, i.e., the promptness of the leader's movement onset on follower's cue to initiate entry. In this crucial time interval, the trial is initiated and both subjects have to ready for their coordinated entry so that the leader is not left exposed alone to potential risks. High leader readiness is associated with a short duration of segment s1 (cf. Table 1). Since a short duration of this time interval indicates that both team members are ready to engage in the task, we use this quantity as a measure for team coordination. During this segment, both participants are immobile (i.e., EEG containing less muscular artifact in the beta/gamma band) yet crucially engaged in the mental effort to coordinate their behavior. In the following we employ the degeneracy framework to identify neural signatures of team coordination. Using leader readiness as a measure of team coordination we analyzed two sets of variables of interest: wavelet phase coherence and wavelet power. These two sets of variables give rise to two state spaces each of which contain two manifolds: The manifold related to high team coordination and the manifold related to low team coordination. In the following section 2.6, we describe the state space of the two measures and in sections 2.7 and 2.8, we discuss the measures and the properties of the manifold that we used to compare joint neural activity of high and low team coordination.

### 2.6. State Spaces of Inter-brain Phase Coherence and Wavelet Power

We start with identifying the state space of brain dynamics for the two measures, phase coherence and wavelet power. The state space of brain dynamics is almost always larger than the behavioral state space, because whole brain coverage (EEG, but also fMRI, fNIRS, etc.) typically involves a large number of measurement sites. Here we used a 60 electrode cap for each subject and perform a wavelet analysis of the EEG data using a complex Morlet wavelet (Bruns, 2004). Complex valued components of oscillatory brain activity have two natural descriptors: amplitude and phase from which power and phase coherence are computed as derived quantities. This yields two potential state spaces: the wavelet power state space and the wavelet phase coherence state space.

Let *k* = 60 be the number of electrodes of one subject and *f* the number of frequencies taken into account. Using wavelet powers as descriptive variables, the dimensionality of the wavelet power state space is *d* = 2*kf* and using phase coherence as descriptive variable the wavelet phase coherence state space dimensionality is *d* = *k*(2*k* − 1)*f*. Here we restricted our analysis to the frequency band of 15 − 40*Hz* (low and high β and low and medium γ band), because the behaviors of interest such as signaling readiness by the follower, movement initiation by the leader, and entry coordination between the two subjects occur at time scales of 200ms or less, corresponding to 3 − 25 cycles in the chosen frequency band. Further we used a frequency resolution of 0.5*Hz* and hence *f* = 51. The state space for wavelet power had hence *d* = 6, 120 dimensions, and the state space for phase coherence had *d* = 364, 140 dimensions. Note that by using proper scaling, the two state spaces could also be combined, but we chose to keep the two descriptions separate.

### 2.7. Inter-brain Phase Coherence

We measured the dynamics of phase coherence using a sliding window of 80 ms (suitable to represent frequencies from 12.5 Hz to the Nyquist frequency and consistent with the length of an EEG microstate; Lehmann et al., 2009). The window was iteratively shifted by 40 ms. Phase coherence in each segment was assessed as the phase locking value (Lachaux et al., 2002)

(1) $$c_{jk}\left(\phi \right)=\frac{1}{N}\left|\sum_{n=1}^{N}\exp\left(\phi_{n}^{\left(j\right)}-\phi_{n}^{\left(k\right)}\right)\right|$$

where *N* is the number of sliding windows in the trial segment and $\phi_{n}^{\left(j\right)}$ and $\phi_{n}^{\left(k\right)}$ are the phase angles of the wavelet coefficients at frequency ϕ at time window *n* and at electrodes *j* and *k*, respectively. Frequencies are ϕ ∈ {15, 15.5, …, 40} Hz and electrodes *j, k* ∈ {1, …, 120}.

For phase coherence the brain dynamics manifold consists of all possible phase coherence trajectories over the time windows in the context of the virtual room clearing task at a given team coordination level. Each point on the trajectory is represented by the coordinates *c*_*jk*_ (ϕ) from Equation (1). The data we have about the brain dynamics manifolds consist of the trajectories that could be constructed from the EEG data. Because of the prohibitively high dimensionality of the phase coherence state space we chose to not explicitly reconstruct the manifold, and instead analyzed the properties of the manifold indirectly via the analysis of its mapping to a lower dimensional space.

To this end we restricted our analysis to intra- and inter-brain phase coherence, respectively. A subject's intra-brain phase coherence in a frequency band for a given trial segment was computed as the average over all phase coherences among all electrode pairs of the subject and averaged over the time windows in the trial segment. Inter-brain phase coherence was assessed in the same way, but instead of averaging over all electrode pairs of one subject, the average was taken over all electrode pairs in which each electrode belonged to a different subject of the dyad. Note that to determine inter-brain phase coherence, all inter-subject electrode pairs were taken into account, not only anatomically corresponding pairs. To determine how intra- and inter-brain phase coherence were related to team coordination we computed the correlations between the team coordination measure of leader readiness (duration of trial segment s1) and the intra- and inter-brain phase coherences in seven crucial segments of the trials, namely the preparatory segments s9, s0, and s1, and the entry segments s2–s5. To avoid spurious correlations due to outliers, we excluded the 5% lowest and 5% highest values for phase coherence and the 5% lowest and 5% highest values for team coordination.

### 2.8. Specific Wavelet Power Patterns in High and Low Team Coordination

As a complementary measure to inter- and intra-brain phase coherence we also analyzed joint wavelet power patterns across the dyad that are specific to high and low team coordination, respectively. Due to the degeneracy of brain dynamics, multiple different dyadic wavelet power patterns could fulfill the specificity requirement. These correspond to the data we have about the manifolds in the wavelet power state space. As with phase coherence we chose an indirect approach to analyzing the properties of the manifold.

To identify joint wavelet power patterns of the dyad that are specific to high and low team coordination, respectively, we divided the data into two classes. The class of high leader readiness included all trials with segment s1 duration < 338 ms and the class of low leader readiness included all trials with segment s1 duration > 523 ms. The cutoff values represent the 35% lowest and highest percentiles taken over all trials and teams and were chosen as a compromise between maximizing the number of trials included in the analysis while simultaneously maximizing the contrast between the two classes. Each dyadic wavelet power pattern can be considered as a vector in a 2 × 60 × 51 = 6, 120 dimensional state space, and our goal is to identify those vectors which are specific for their respective class. As mentioned above, due to the degeneracy of brain processes various distinct vectors could fall into the same class. To determine class specific dyadic wavelet power patterns, we computed the distance matrix between all dyadic wavelet power patterns (each considered as a 6,120-dimensional vector) in the high and low team coordination class. The distance between two dyadic wavelet power patterns **x** and **y** was thereby defined as

(2) $$\begin{array}{l} d\left(\text{x},\text{y}\right)=\frac{\|\text{x}-\text{y}{\|}^{2}}{\left\|\text{x}\|\|\text{y}\right\|} \end{array}$$

Class specific dyadic wavelet power patterns were then computed as follows: Let *N* and *n* be the numbers of dyadic wavelet power patterns of high and low team coordination, respectively. Then the distance matrix is an (*N* + *n*) × (*N* + *n*) matrix with the square *N* × *N* and *n* × *n* blocks on the diagonal containing the respective intra-class distances (high and low team coordination respectively) and the *N* × *n* and *n* × *N* rectangular off-diagonal blocks containing the respective inter-class distances. Due to the symmetry of the distance matrix, the two off-diagonal blocks are the transpose of each other. To define intra- and inter-class distances we performed an ordering of the values in each column of the square and rectangular blocks by increasing distance, respectively. After such a reordering each column of a block represents the distance between the dyadic wavelet power pattern indexed in this column and all other dyadic wavelet power patterns in increasing order, separately for intra- and inter-class distances. We defined as intra-class distance the 10-th percentile row of the ordered square blocks, and as inter-class distance the 10-th percentile row of the ordered rectangular blocks. The 10-th percentile was chosen to keep the intra-class distances small while avoiding outliers affecting the distance measure. A dyadic wavelet power pattern was deemed specific to high or low team coordination, respectively, if its inter-class distance was greater than the intra-class distance. Note that this method can be used to identify specific patterns for any two classes and definitions of pattern vectors. For instance, it could be used to determine phase coherence patterns in high and low performance trials. The advantage of this method is that it can yield specific patterns even for highly overlapping classes. Furthermore, using a percentile as the distance measure rather than the maximum distance prevents the results from being dominated by outliers.

Because of neural degeneracy the specific dyadic wavelet power patterns are expected to be diverse. To assess the similarities of dyadic wavelet power patterns between different electrodes we calculated the *k* × *k* correlation matrix for the *k* = 60 electrodes of each team member role (leader and follower, respectively). Correlation was assessed across frequencies and the dyadic wavelet power patterns over all subjects in the respective role. We then applied a spectral clustering method to reorder the two resulting correlation matrices in such a way that highly correlated clusters appear as square blocks along the diagonal. This allows to identify electrodes with similar wavelet power patterns within each team role (leader and follower, respectively).

To determine how the *f* = 51 frequencies in the specific wavelet patterns in leader and follower were related to each other, we averaged the specific dyadic wavelet power patterns of the medial centro-parietal electrodes and computed their inter-subject correlation matrix across the various specific wavelet power patterns. This yielded two *f* × *f* correlation matrices, one for the high and one for the low team coordination condition. We then defined a coefficient of asymmetry for each matrix element and performed a Wilcoxon signed rank test on the asymmetry coefficients of both matrices.

## 3. Results

Consistent with the conceptual framework of degeneracy we identified multiple signatures of team coordination in the brain dynamics of the dyads, measured by EEG and analyzed by wavelet phase coherence and wavelet power patterns, respectively.

### 3.1. Inter-brain Phase Coherence Correlated With Team Coordination

**Figure 3:**
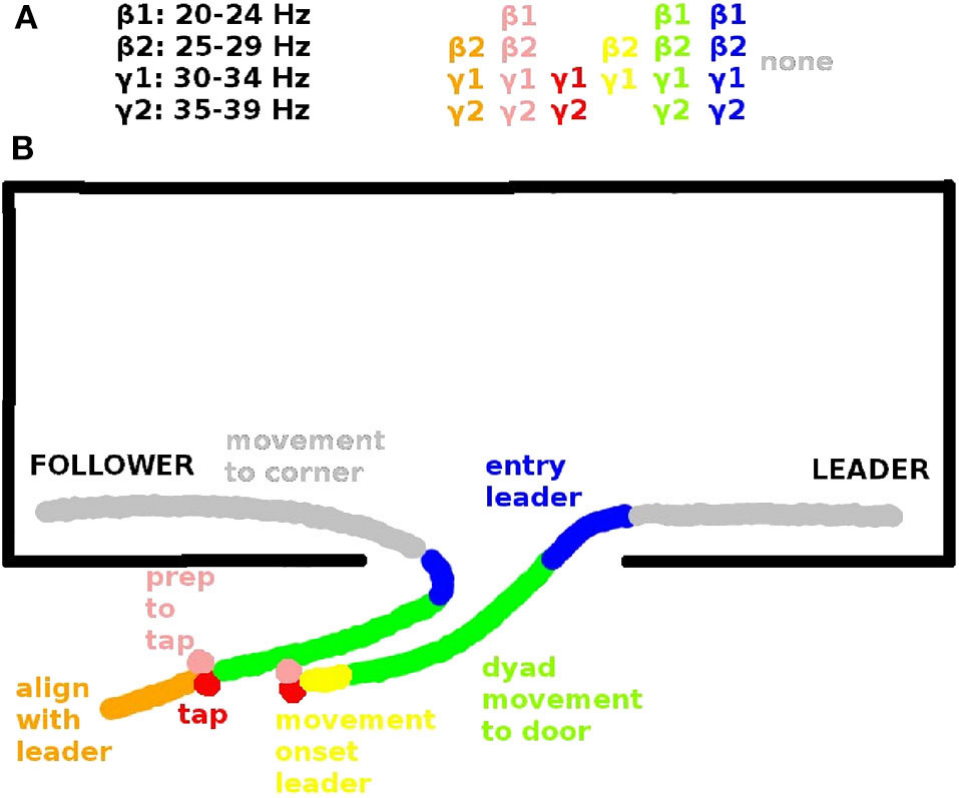
**(A)** Inter-brain coherences in the β and γ range that are significantly correlated with leader readiness and **(B)** corresponding spatial view on an exemplary behavioral sequence. Behavioral sequences in each trial include: Alignment of follower to leader (orange, segment s9, see Tognoli et al., 2011a), preparation to tap (pink, s0), tap (red, s1), movement onset leader (yellow, s2), dyad movement to door (green, s3), entry (blue, s4), movement to corners of dominance. (gray, s5). The significant inter-brain coherences (top) are color-coded according to the segments in which they are significant (see also Tognoli et al., 2011a).

In our data inter-brain phase coherence in the β (20–29 Hz) and γ band (30–39 Hz) showed a significant positive correlation with leader readiness, the measure we used for team coordination, in the segments that involved information exchange between the subjects (cf. Table 2), but not in the segment of moving to the corners of the room, which did not involve inter-subject information exchange. Figure 3 summarizes the trial segments and the frequency bands in which inter-brain phase coherence was correlated with leader-readiness. Interestingly, intra-brain phase coherence was not significantly correlated with leader-readiness, neither in the leader, nor in the follower. In our conceptual framework, this finding indicates that team coordination reduces the dimensionality of the manifold by constraining the brain dynamics between subjects. Another intriguing aspect of this result is that inter-brain phase coherence was significantly correlated with leader readiness also in segments prior to segment s0 from which leader readiness was measured. In particular inter-brain phase coherence in segment s9, where the avatars of both subjects move to the next stacking point and in segment s0 when the leader waits for the tap of the follower. While the well-known caveat that correlation does not imply causation of course still holds, this sequence of events indicates that increased inter-brain phase coherence during restacking and tap preparation leads to higher leader-readiness.

**Table 2:** Correlation of inter brain phase coherence with leader readiness.

**Preparatory segments (Hz)**	**s9**	**s0**	**s1**	
	**Alignment**	**Preparation to tap**	**Tap to movement**	
20–24	0.0700	0.1072^**^	−0.0255	
25–30	0.0890^*^	0.1325^***^	0.0574	
30–34	0.1188^**^	0.1270^***^	0.1112^**^	
35-39	0.1142^**^	0.0898^*^	0.1525^***^	
**Entry segments (Hz)**	**s2**	**s3**	**s4**	**s5**
	**Movement leader to movement follower**	**Movement to door**	**Entry**	**Movement to corners**
20–24	−0.0122	0.0917^*^	0.0913^*^	0.0099
25–30	0.0907^*^	0.0784^+^	0.1006^**^	0.0699
30–34	0.1294^***^	0.1067^**^	0.1445^***^	0.0453
35–39	−0.0063	0.0942^*^	0.1157^**^	0.0750

+, ^*^, ^**^, and ^***^ indicate significant correlations at p < 0.05, p < 0.02, p < 0.01, and p < 0.001, respectively.

### 3.2. Team Coordination Related to Asymmetry of Information Transfer Reflected in Wavelet Power Patterns

Team coordination does not necessarily involve similar processes in the two brains of the dyad. Particularly in trial segments with unidirectional information flow such as when the follower has to coordinate his or her avatar's behavior to the leader's, but not vice versa, e.g., in the entry segment (s4), or in segment s1 where the follower signals the leader to initiate movement, we expect different brain activity patterns in the two subjects (s0–s4), depending on their respective role as leader or follower. We hence asked the question whether there were specific dyadic wavelet power patterns that were associated with high or low team coordination, respectively. Here we concentrated on segment s1 where team coordination occurs in terms of leader readiness. As expected from our discussion about behavioral variability and degeneracy of brain processes, we found multiple dyadic wavelet power patterns that were associated with high and low leader readiness, respectively, in 14 out of 16 dyads. Spectral clustering revealed a set of medial centro-parietal electrodes that showed very similar wavelet power patterns within each team role (leader and follower, respectively). This set was essentially the same in the leader and the follower and in the low and high leader readiness conditions, and included a wide territory over the medial aspect of posterior cortex (electrodes C1, Cz, C2, CP1, CPz, CP2, P3, P1, Pz, P2, and POZ). This means that while the dyadic wavelet power patterns that were specific to high and low team coordination did exhibit degeneracy, the individual wavelet power patterns were highly similar across a set of 11 centro-parietal electrodes, hence indicating that both brain dynamics manifolds, corresponding to high and low team coordination, on which the specific dyadic wavelet power patterns “live,” have reduced dimensionality.

**Figure 4:**
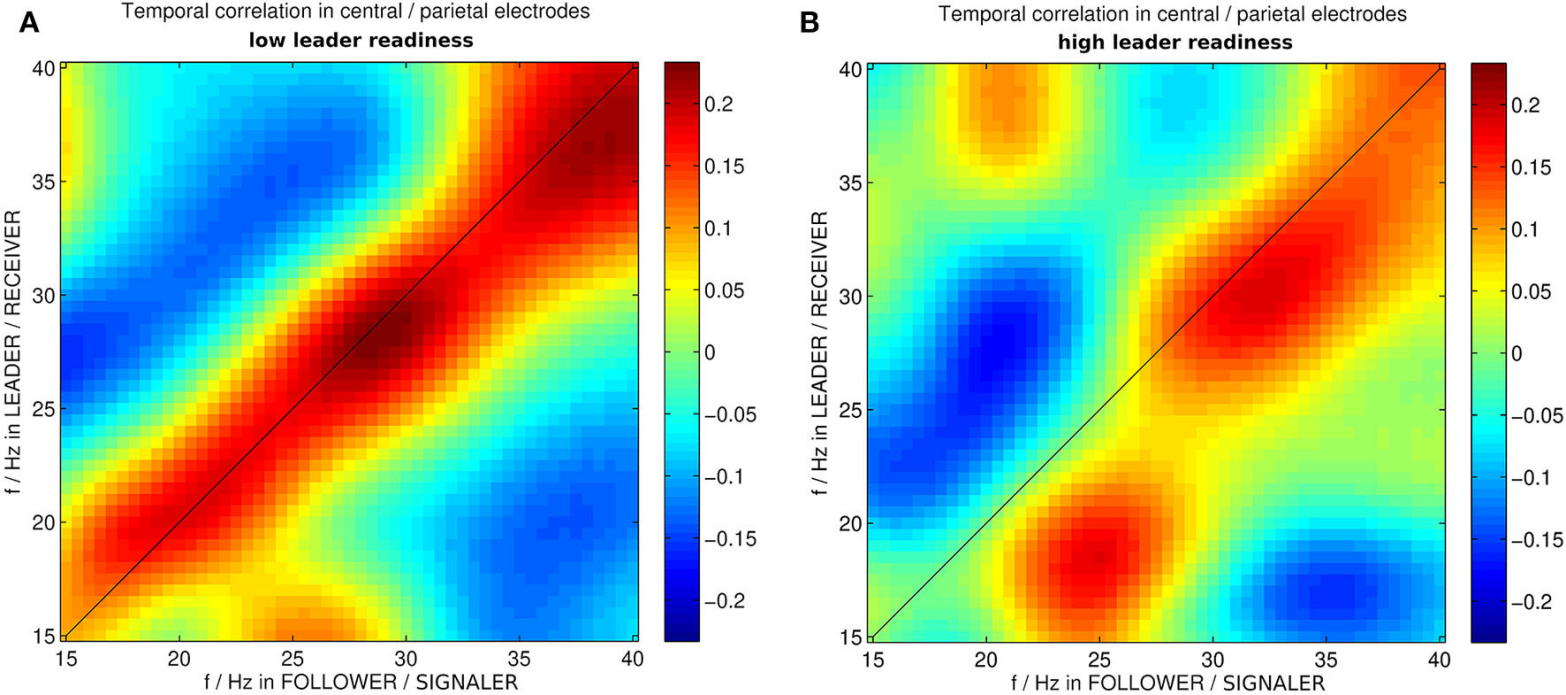
Correlation between wavelet powers in medial centro-parietal electrodes of leader and follower in instances of dyadic wavelet power patterns specific to **(A)** low and **(B)** high leader readiness, respectively. *f*/*Hz* stands for frequencies in Hz. The different roles in this segment are the follower acting as signaler and the leader acting as receiver.

Furthermore the specific wavelet power patterns in these medial centro-parietal electrodes exhibited an asymmetry in the high team coordination correlation matrix for different frequencies (cf. Figure 4). The correlation matrix corresponding to high leader readiness (as a measure of high team coordination) was significantly more asymmetric than the correlation matrix corresponding to low leader readiness. In trials with low leader readiness similar frequencies in the centro-parietal electrodes of the leader and the follower tended to occur together (cf. Figure 4A). By contrast we found that in trials with high leader readiness only frequencies above 25 Hz tended to occur together in the centro-parietal electrodes of leader and follower. Lower frequencies were uncorrelated and two patterns of asymmetric correlations between different frequencies in leader and follower occurred: High β power (25 Hz) in the follower tended to occur together with low β power (18 Hz) in the leader, and low β power (20 Hz) in the follower tended to co-occur with low γ power (30 Hz) in the leader. This asymmetry reflects the uni-directional information flow from follower to leader in this segment (s1) and indicates that an asymmetry in the brain dynamics of the dyad, corresponding to their respective roles, is associated with efficient information transfer (high leader readiness) in this segment.

## 4. Discussion

In our quest for neural signatures of team coordination we proposed a general geometric framework that addresses degeneracy in behavioral and brain dynamics. Degeneracy is an inevitable attribute of team coordination in realistic settings and an important property of biological systems (Whitacre and Bender, 2010). It occurs at multiple spatio-temporal levels, from biological networks (Beverly et al., 2011) to human movement (Kelso et al., 1998, 1980b; Davids and Glazier, 2010). Hence for the advancement of our understanding of team coordination and more generally the behavior of biological systems there is an urgent need for methodologies that allow the analysis of processes involving degeneracy. Our framework is a first step in this direction. By parameterizing the behavioral and brain dynamics as trajectories in their respective state spaces and interpreting their ensemble as manifolds, our framework relates degeneracy to geometry thereby making geometrical methods available for the analysis of tasks that involve degeneracy. Rather than viewing variability as nuisance, our framework makes use of the variability that originates from degeneracy to identify crucial properties of behavioral and brain dynamics, including task constraints, team performance, and team coordination. The close relationship between the geometry of the manifold and the task-related behavioral properties can be used to infer information about these properties, e.g., to track the dynamics of team coordination (Dodel et al., 2010) or identify neural signatures of team performance (Dodel et al., 2011). Tasks involving degeneracy include but are not restricted to team coordination tasks, in fact any complex task with a high number of degrees of freedom is prone to exhibit degeneracy. Importantly, degeneracy challenges the quest for the identification of a single underlying mechanism of a behavior in a biological system, as there may be multiple mechanisms that support the same function (Whitacre, 2010; Kelso and Tuller, 1984). Our finding of multiple specific dyadic wavelet power patterns is consistent with this view. Moreover, efficient unidirectionality of the information flow in trial segment s1 (Figure 4) concurs with an observed symmetry breaking of co-occurring frequencies, reinforcing the notion of specific, but not unique dyadic wavelet power patterns being associated with elements of team coordination. Our results further indicate that inter-subject coordination is associated with phase coherence in the β and γ bands between the two brains of the dyad, but not within brains.

Our findings support the increasing evidence that inter-brain phase coherence plays a role in social interaction (Dumas et al., 2010; Cui et al., 2012; Dommer et al., 2012; Jiang et al., 2012; Sänger et al., 2012; Yun et al., 2012; Kawasaki et al., 2013). Obviously, inter-brain phase coherence cannot be mediated by the same mechanisms as intra-brain phase coherence since there is no anatomical connectivity between the two brains. Rather inter-brain phase coherence is associated with an inter-personal perception ~ action cycle and reflects the information flow between the subjects as they act together as a functional unit. While some might argue that our results were primarily due to shared visual input we deem this unlikely as one would expect the intra-brain coherences to be correlated, too, but the correlations occurred only for inter-brain coherences. There could be an effect of attention (Lachat et al., 2012) as after the entry the team members may encounter enemies and may direct their individual attention to this threat. Inter-brain phase coherence might be favored by joint perceptual experiences (Hasson, 2004) and symmetries in the human brain's anatomical connectivity grounded in a shared phylogenetic and ontogenetic path (Bressler and Tognoli, 2006; Tognoli, 2008; Dumas et al., 2012; Tognoli and Kelso, 2015). Furthermore, inter-subject phase coherence could be a neural signature of team cognition (Fiore and Salas, 2004), i.e., of the subjects forming a cognitive unit during efficient social coordination. Further studies and models of a dyadic (and eventually polyadic) perception-action cycle are needed to further elucidate the role of inter-brain phase coherence in social contexts.

Team coordination involves different behaviors of the two subjects throughout the trial as summarized in Table 1. The asymmetry of wavelet patterns in tasks with high leader readiness may be the reflection of the directionality of the information flow in segment s1 in the brain dynamics of the dyad. More specifically, the leader is the receiver of the tap and hence increased leader readiness may be associated with increased attention and motor preparation in the leader. These processes may be mediated by increased γ power (Müller et al., 2000; Bauer et al., 2006), and increased low β power (Kristeva-Feige et al., 1993) in the leader and associated with increased β power in the follower after finger movement to apply the tap (Pfurtscheller et al., 1996). In tasks with lower leader readiness, these processes may not occur simultaneously, leading to a longer time interval between tap and movement onset. The symmetry of wavelet patterns in trials with low leader readiness may indicate further brain processes that hamper the processing of directional information flow. Performance during this segment hinges on the two participants playing distinct roles. Therefore, symmetry of the wavelet patterns is likely related to a failure to differentiate the role requirements that the task demands from both participants.

Behavioral studies in movement sciences have a history of dealing with degeneracy (Kelso et al., 1980b; Kelso and Tuller, 1984; Scholz and Schöner, 1999). Degeneracy has been found to be an important asset to achieve motor control in systems with many degrees of freedom (Todorov and Jordan, 2002). We argue that degeneracy is a multi-level phenomenon, extending from single subject to group behavior and from the behavioral to the neural regime (e.g., Kugler et al., 1980; Kelso et al., 1980b; Todorov and Jordan, 2002). The framework presented here conceptually elucidates the relation between different levels and hence allows for a multi-level description of group processes.

Using the present framework, we have identified neural signatures of inter-subject coordination in an ecologically valid task using two sets of variables of interest. The choice of variables, and hence the choice of the state space in which the respective behavioral and brain dynamics are described, is an *a priori* one, guided by the research question and the data at hand. Here we have used wavelet power and phase coherence owing to the oscillatory nature of brain activity and its reflection in the EEG. Other brain imaging methods may require other choices of variables of interest. The experimental data represent a sample of the underlying manifold and hence inference of the geometry of the manifolds from the data may require a low-dimensional state space or the mapping of the manifold onto a low-dimensional one. Interestingly, this is not such a strong restriction as it might seem, since due to synergies, coordination is often associated with low dimensional dynamics (Kelso, 1986; Schöner and Kelso, 1988). Our finding of increased inter-subject phase coherence indicates that similar mechanisms may be at work in the brain. The framework presented here allows further generalizations. For instance, while degeneracy allows multiple behavioral or brain dynamics to accomplish a given task, certain dynamics may be more frequent than others. This can be accommodated by defining a probability density on the manifold which can be estimated by the density of state space trajectories. Furthermore, the state space trajectories define a directionality or flow on the manifold. Our framework also can incorporate learning by assessing manifolds at different stages of expertise in a task and tracking their evolution. Finally, we are not suggesting that all variability is due to degeneracy: for example, some variability may be noise related. However, we expect the latter variability to be much smaller than the former.

To summarize, the present conceptual framework provides a unifying account for tasks with multiple degrees of freedom and hence is particularly suited to guide the analysis of team tasks in the presence of degeneracy. Degeneracy is a way for biological systems to cope with the ancient doctrine of Heraclitus that no one can step into the same river twice. In accounting for degeneracy our framework represents a departure from the quest for single neural mechanisms underlying given behaviors and explicitly acknowledges the highly synergistic nature of brain processes.

## Data Availability Statement

The datasets generated for this study are available on request to the corresponding author. Wavelets and software related to this study can be found at https://gitlab.com/BrainyDays/Degeneracy.

## Ethics Statement

The studies involving human participants were reviewed and approved by IRB at Florida Atlantic University. The patients/participants provided their written informed consent to participate in this study. Written informed consent was obtained from the individuals for the publication of any potentially identifiable images or data included in this article.

## Author Contributions

ET and JK designed the study. SD analyzed the data. All authors prepared the manuscript.

## Conflict of Interest

The authors declare that the research was conducted in the absence of any commercial or financial relationships that could be construed as a potential conflict of interest.
